# Process Optimization for Acid Hydrolysis and Characterization of Bioethanol from Leftover Injera Waste by Using Response Surface Methodology: Central Composite Design

**DOI:** 10.1155/2022/4809589

**Published:** 2022-04-06

**Authors:** Abreham Bekele Bayu, Temesgen Abeto Amibo, Surafel Mustefa Beyan

**Affiliations:** School of Chemical Engineering, Jimma Technology Institute, Jimma University, Jimma, Ethiopia

## Abstract

In this study, leftover injera waste from the southwestern parts of Ethiopia was used as a raw material for bioethanol production. The conversion of this biomass into ethanol involved processing techniques, which include hydrolysis, fermentation, and distillation. This research focuses on determining optimal parameters that are temperature, acid concentration, and hydrolyzing time in a hydrolysis stage. Using response surface analysis, the suggested model is quadratic and has three independent factors, which had significant effects on the yield of ethanol. In this analysis, the temperature and hydrolyzing time had a positive relationship with the yield of ethanol whereas acid concentration had a negative relation. The optimum yield of ethanol obtained was 79.07%. The yield optimized in g/g was 29.99, which was obtained at a temperature of 109.99°C, at an acid concentration of 1.00%, and hydrolyzing time of 49.59 minutes. For this analysis, the mathematical model equation was developed and the *R*^2^ value was 99.9% and its desirability was 0.8867. The property of ethanol was characterized by the many parameters used in different standardization. The density, viscosity, flammability, boiling points, and pH were determined as 0.803 gcm^−3^, 1.1 cP, 14°C, 80°C, and 6.65, respectively.

## 1. Introduction

The current energy demand for fossil fuels is increasing dramatically; also, it is the dominant source of energy in the world. The world has high demands towards energy to satisfy daily activity; for this reason, the energy demands are increasing day to day [1]. The world energy demand is satisfied by fossil fuel it is a nonrenewable energy source; once it is depleted, it is not replaced again [2]. The main drawback of fossil fuels is generating greenhouse gases (GHGs). The GHGs result in the environment becoming warmer and causing unpredictable and dramatic changes in climatic conditions [3, 4]. The limited availability of renewable energy sources leads to environmental problems. Such negative impacts of fossil fuels on the world have driven the globe's attention towards sustainable energy sources [5]. To alleviate such problems, another energy source is focused on; such energy is a renewable energy source. World energy demands for machinery, vehicles, and others are increased; then to meet such requirements, additional energy source is needed to satisfy the energy demands [6].

Thus, it is better to focus on the renewable energy source like bioethanol from waste biomass, which is leftover injera waste [7]. Nowadays, the demand for bioethanol is an increase in its volume and consumption [8]. Ethiopia generates about 192,000 metric tons of leftover injera waste annually, from those around 134,400 metric tons of waste was generated in the southwestern region of Ethiopia especially around Jimma town [9]. The leftover injera waste is a waste of raw material that is disposed to the environment from restaurants and student cafeteria wastes. This leftover injera waste is abundant biomass present in Africa, especially in the southwestern parts of Ethiopia [10]. The leftover injera waste is simply dumped on the open land surface without any treatments. Such damp waste causes water and environmental pollution [11]. Due to a massive accumulation of leftover injera waste, sometimes it generates and causes a fire on forests in a hot season. Rather than disposing of this biomass (leftover injera waste), it is better to convert it to bioethanol [12]. Converting this leftover injera waste into bioethanol has two major benefits. The first one is keeping our environments sustainable; this helps to keep biodiversity. The second benefit is converting this waste to energy, that is, saving the raw material costs and replacement of nonrenewable energy (like coal, crude oil, and natural gas) with renewable one [13].

The optimization process of the acid hydrolysis for bioethanol production was analyzed by using surface response methodology with the design expert [14–16]. Acid hydrolysis results in higher yields of simple sugar than the other enzymatic hydrolysis. Acid hydrolysis has a good reproducibility compared to other methods; it is very common and cheapest if compared with the other methods of the hydrolyzing process [17]. The 5-level 3-factor (i.e., temperature, acid concentration, and hydrolyzing time) experiment was designed by the central composite design (CCD) method using a Design-Expert (version 11) [18]. Experiments were conducted according to the designed process conditions. Furthermore, numerical optimization was carried out using the response surface method (RSM) to maximize bioethanol yield and physicochemical properties of produced bioethanol were determined and compared to bioethanol standards.

From the previous studies, it was observed that there were no researches that were done for the optimization of acid hydrolysis on ethanol production from leftover injera waste. Thus, the main objective of this study is to optimize the acid hydrolysis process for bioethanol production from leftover injera waste. The optimal points of each parameter like temperature, acid concentration, and hydrolysis time during hydrolysis condition were determined. Finally, the determination of bioethanol fuel quality was justified based on characterization of ethanol's density, pH, viscosity, FTIR analysis for functional group determination, flammability, boiling points, and the alcohol contents of bioethanol produced in a laboratory scale, and so on.

## 2. Methodology

### 2.1. Material and Chemicals

#### 2.1.1. Materials Used

The experiment was handled at Jimma University Institute of Technology in the chemical engineering laboratory. The different production and characterizing equipment were used during the experiment. Digital ovens were used to measure moisture content. Alcoholmeter is used to determine the alcohol content in a produced ethanol. The autoclave was used for sterilization and hydrolysis of the leftover injera waste sample during the breakdown of large components to small ones. The autoclave has a thermostat to control temperature during the hydrolysis process. Balances, incubators, and distillation were used for the purification of ethanol from a solution. A digital pH meter was used to measure the pH of acids as well as ethanol. Sieves, viscometer, magnetic stirrer, aluminum foil, crushers, and fine cloth were used.

#### 2.1.2. Chemicals Used

H_2_SO_4_ (sulfuric acid), initially 98%, was used; this acid concentration was diluted during acid hydrolysis. Dry instant yeast, ammonia solution with a concentration of 28%, dextrose (C_6_H_12_O_6_) or glucose, distilled water, hydrochloric acid (HCl), and sodium hydroxide (NaOH) were used during pH adjustment.

### 2.2. Methods

Leftover injera waste is available in the southwestern parts of Ethiopia. Especially, for this study, Jimma, Bonga, and Bita areas were selected for the collection of raw material as well as the data. The sample collected was transported to Jimma University Chemical Engineering Laboratory for further process. This leftover injera waste was dried to remove the water content by putting it in the oven. The oven's temperature was maintained at 65°C for three days. The dried leftover injera waste was ground to small size by using mortar and pestle. Then, it was put into dry place far from moisture. The potential of leftover injera waste is high and abundant in Ethiopia.

### 2.3. Sample Collection and Preparation

#### 2.3.1. Sample Grinding

**Figure 1:**
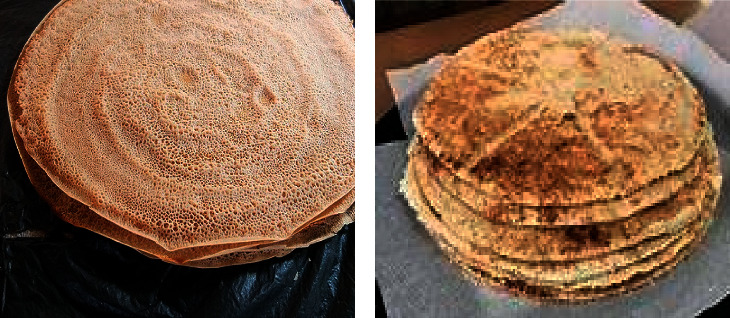
Injera. (a) Normal injera. (b) Leftover injera waste [19].

According to [19], the sample was dried within a specified time. As shown in Figure 1(b), the dried leftover injera waste was crushed by using mortar and pestle to appropriate size for the experiment. The maximum particle size of the ground mixed sample was 3 mm. The grounded sample was kept far away from the availability of moisture and allowed to stay at room temperature.

#### 2.3.2. Sample Screening

In the study of [20], the ground sample was separated based on its size by using screening methods. By using different sieve sizes, the coarse-sized sample was removed. The required size of the sample was 2 mm; the sample greater than 2 mm was retained on the sieve from the analysis. For the acid hydrolysis process, the size of the sample selected from 2 mm to 3 mm has a high yield of ethanol [21].

### 2.4. Laboratory Procedures

In this section, the experiments were carried out to produce bioethanol from leftover injera waste. From Table 1, the chemical composition of leftover injera waste was analyzed by standard methods like the American Society for Testing and Materials (ASTM). Also, in this study, the cellulose, hemicellulose, lignin, ash content, and extractives were analyzed. The concentrated sulfuric acid 98% was diluted to a different concentration that was analyzed by CCD. After being dried and ground, the screened sample was weighed based on the proportion of sample to solvents. The weight of the sample was 41 g for all experiments carried out. This sample was added into an acid solution of Erlenmeyer flask (500 mL) with a concentration of 0.66%, 1%, 1.5%, 2%, and 2.3%. The acid hydrolysis process was carried out in an autoclave digester that has a thermostat with a capacity of 15 L and which has equipped with a heater, digital temperature, and pressure controller. The pressure for this experiment was maintained at 1 bar. The hydrolyzed sample was taken from the autoclave and filtered by using a fine cloth and filter paper to increase its purity. Before the fermentation process, neutralization process was carried out to maintain a solution pH of 6.5. This helps the growth of microorganisms during the fermentation process. In the sample, the yeast (saccharomyces cerevisiae) was added to speed up the fermentation process. Then, the hydrolyzed sample was placed within an incubator at a temperature of 31°C for 72 hours. The fermented sample was taken from an incubator and purified using fractional distillation at a temperature of 79°C.

### 2.5. Experimental Design

Design-Expert (11.0) software was used to analyze the experimental results. Response surface methodology is the best technique used for the optimization process of the independent variable [22, 23]. It is also used to determine the factor which affects the yields of ethanol in a better manner. This helps to determine which factor is significantly affecting the yield and insignificantly affect the yields. In this response surface methodology, central composite design (CCD) was selected to determine linear, interaction, and quadratic of the independent variable during hydrolysis of polysaccharides to fermentable sugar. The RSM generates actual data points, axial data points, and center data points [24]. This helps to predict the interaction effects of each factor; the data points were selected based on different works of literature and the selected ranges were highly affected by acid hydrolysis. In this study, three factors were independent variables and others held constant. These were temperature, acid concentration, and hydrolyzing time. By using CCD 5-level, 3-factor, and 20 data points are generated based on the equation (1) including 8-factor points, 6-axis points, and 6 points as a center.

(1) $$\begin{array}{c} N=2^{n}+2n+n_{c}=8+6+6=20, \end{array}$$

where *N* is the number of experiments being carried out, *n* is the number of the independent variables, and *n*_*c*_ is the number of replicates used to check whether the above-conducted experiment was either correct or incorrect.

### 2.6. Raw Material and Product Characterization

Proximate and ultimate analyses of leftover injera waste according to the study [25]. In this study, the proximate analysis was investigated according to ASTMD methods. The moisture content, volatile matter, ash, and fixed carbon were analyzed and investigated. The chemical composition of leftover injera waste was analyzed by using different methods compositions like cellulose, hemicellulose, lignin, protein content, lipids, and other organic material found within leftover injera waste. According to [26], bioethanol was characterized. According to this study, bioethanol was characterized by its density, flammability, viscosity, boiling points, pH, alcohol content, and its functional group analysis by using FTIR analysis. The produced ethanol was checked for its functional group by using Fourier-transform infrared spectroscopy. After that, the other parameters were checked.

## 3. Result and Discussion

### 3.1. Characterization of Raw Materials

The raw material of leftover injera waste was characterized according to ASTMD methods. According to the study of [27], the proximate analysis for leftover injera waste was analyzed. The carbohydrate content was 58–85% (this constitutes cellulose, hemicellulose, and lignin parts), the protein content of 8–11%, the lipid content of 0.5–3%, the mineral content of 3–7%, and caffeine content around 1%. Proximate analysis for leftover injera waste was analyzed to determine the cellulose, hemicellulose, lignin, ash content, and extractives by using standard methods of ASTMD. Besides this, leftover injera waste ultimate analysis was also carried out; for example, moisture content, ash content, and fixed carbon content were characterized. From the study of [28], the proximate analysis of leftover injera waste was analyzed and clearly described. In this study, analyzed leftover injera waste was leftover injera waste, which was similar to [29]. The cellulose and hemicellulose were obtained by using ASTM D 5896-96 (2019) methods. The results obtained were 26.2% and 25.6%, respectively. The lignin portion was analyzed by ASTM D1106 (2013) method, and then the obtained result was 33.8%. The extractives were analyzed by ASTM D1105-96 (2013) methods and their value was 6.6%. There is an insignificant deviation between the values obtained from [30]. This wet bases leftover injera waste has a moisture content determined by digital oven was 12%. According to [31], the moisture content for the dry basis of leftover injera waste was 8.8%; this shows that the leftover injera waste has high moisture content compared with previously done. The volatile matter for this study was 82.15%; compared with the previous study, it has less amount of volatile matter. The previous study volatility was 83.24%, which indicates that it is higher than that of this study. The ash content of leftover injera waste is 1.1%; compared with the previous study, the current study constitutes high ash content. The fixed carbon content of this study was 16.75; this is shown in Table 2.

### 3.2. Statistical Data Analysis

From Table S1, the statistical data analyzed by using Design-Expert (ANOVA) is as shown below. For this study, the level of significance (*α*-value) was 5%, and this helps to determine the significance of each factor. Probability (*p*-value) obtained from the central composite design matrix has helped to determine the significance of each factor and the interaction effects by comparing with the level of significance. When the *p*-value is less than the *α*-value, the factors (independent variable) have a significant effect on the response [32]. From Table S2, the model is significant, which means its *p*-value was less than (*α*-value) and the suggested model was accepted. The *p*-value of the model was less than 0.0001, which means significant. All factors temperature (A), acid concentration (B), hydrolyzing time (C), AB, BC, AC, A^2^, B^2^, and C^2^ had a significant effect on the yield of ethanol. That means the *p*-value analyzed by CCD was less than the level of significance, that is, 0.05. The statistical data analyzed from ANOVA the regression model was found to be highly significant from Table S3. The *R*^2^ value obtained from the analysis is 99.8%, which means the model fitted very well with the experimental value. This indicates that the model was highly correlated with the experimental values. Also, the *R*^2^ value indicated that the predicted and actual values were fitted very well.

Table S2 represents the significance of single factors and interaction effects on the yield of ethanol obtained based on the values of the level of significance. The model suggested by ANOVA was quadratic, and all single factors have significant effects on the ethanol yield obtained. The *p*-value of temperature, acid concentration, and hydrolyzing time was <0.0001, <0.0001, and <0.0001, respectively. This *p*-value of A, B, and C was less than the *α*-value; this shows that all single factors had a significant effect on the yield of ethanol. In the same way, interaction effects such as AB, AC, and BC have a *p*-value of <0.0001, 0.0003, and <0.0001, which were less than *α*-value (0.05); this implies that these three interaction effects had a significant effect on the yield of ethanol. Also, A^2^, B^2^, and C^2^ had significant effects on the yield of ethanol, which means its *p*-value is less than the *α*-value. The lack of fit indicates that the model of regression is good or poor; it is analyzed by the lack of a fit model. The *p*-value of lack of fit is less than the *α*-value; the model of regression is poor and the *p*-value of lack of significant model was greater than the *α*-value; the model represented regression in a good manner [33]. The lack of fit model is insignificant, which means its *p*-value is greater than the *α*-value. The sum of square values is used for the determination of which factor highly affects the yield of ethanol. The sum square value is higher, which means the factors affect the yield sign and less value indicates that factors affect less significantly than the higher [34].

### 3.3. Developing of Regression Model Equation

From Table 2, of the fit summary, the following were obtained from ANOVA: each value of std. dev., mean, C.V%, *R*^2^, adjusted *R*^2^, and predicted *R*^2^. Precision and the model suggested were quadratic. According to [35], the adjusted *R*^2^ and predicted *R*^2^ should be deviated within 20% to be in good agreement. The adjusted *R*^2^ and predicted *R*^2^ for this study were 0.9952 and 0.9981, respectively. The adjusted *R*^2^ and predicted *R*^2^ describe many independent variables to judge its models. Most of the time predicted *R*^2^ value is smaller than *R*^2^; this indicates that warnings sign that the model is overfitting. Sometimes adjusted *R*^2^ and predicted *R*^2^ values are equal when the model is perfectly fitted and the *R*^2^ value is one. It satisfies the above requirements stated. The model suggested was quadratic and highly significant; it indicated the good agreement between the experimental results obtained and the predicted value obtained from the response surface analyzed. The percent of the coefficient of variation and the standard deviation is low; it is acceptable for analysis [36]. The percent of the coefficient of variation and standard deviation for this study were very low; they were 0.4412 and 0.1166, respectively. These values are very low and acceptable. Both the standard deviation and coefficient of variation are measured by the relative dispersion of all data points that the sample was taken. Coefficients of variation and standard deviation values are low, meaning all data points are close to each other and small deviation between data points occurs. Also, the data obtained and regression values were precise to each other and the fitting model was good [37]. The *R*^2^-values (0.998) indicate that the experimental results and predicted values were very precise.

The mathematical model equation of ethanol yields was developed by using coded variables of each factor. From Table 2, the coefficients of each coded variable were obtained. This result also helps to predict which factor was affecting the ethanol yield positively and negatively. Positive coefficients affect the yield of ethanol positively and whereas negative coefficients affect the yield of ethanol negatively. The coded factors A, C, A^2^, and B^2^ have positively affected the yield of ethanol whereas B, AB, AC, BC, and C^2^ had negatively affected the yield of ethanol. The intercepts for this equation were 26.75, which helps to predict the precise results of ethanol yield. The other coded variables have coefficients determined for A, C, A^2^, and B^2^ were 1.70, 1.67, 0.4419, and 0.1035, respectively. The factors that affect the yield of ethanol negatively were B, AB, AC, BC, and C^2^. Then, their values were −1.30, −0.5650, −0.2200, −1.19, and −1.02, respectively. Based on this, the mathematical model developed is represented in equation (2). When the effect of factors A, C, A^2^, and B^2^ increases, the yield of ethanol was increased proportionally within the specified range. In the same way, when the effect of factors like B, AB, AC, BC, and C^2^ increases, the yield of ethanol was decreased. The coded variable was used for model equation development of bioethanol because it is simple to represent the equation and simple to understand. The temperature (A) of the hydrolysis process increasing the yield of ethanol was increased. This reaction is the endothermic reaction: when the temperature increases, the formation of the product is favored. When the acid concentration (B) increases, the yield of ethanol was decreased due to lower diffusion rate within the solution. The time of hydrolysis has increased; the yield of ethanol was increased. This is due to the polysaccharides having enough time to break down into simple sugar [38].

(2) $$\begin{array}{c} Y=26.75+1.70A-1.30B+1.67C-0.5650\text{AB}-0.2200\text{AC}-1.19\text{BC}+0.4419A^{2}+0.1035B^{2}-1.02C^{2}. \end{array}$$

### 3.4. Response Surface Analysis for the Ethanol Yield

**Figure 2:**
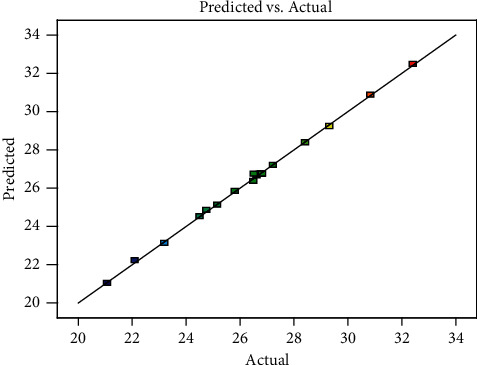
The predicted and actual value.

Predicted and actual values were very close and they are represented by *R*^2^ values. *R*^2^ -values were 99.9% as shown in Table S3 analyzed by analysis of variance. This result indicates that the predicted values and experimental values are comparable as well as very close to each other. The model suggested and the fit of the line to data points are highly accurate. The predicted and actual values are represented in Figure 2.

### 3.5. The Single Factors and Interaction Effects on Ethanol Yield

**Figure 3:**
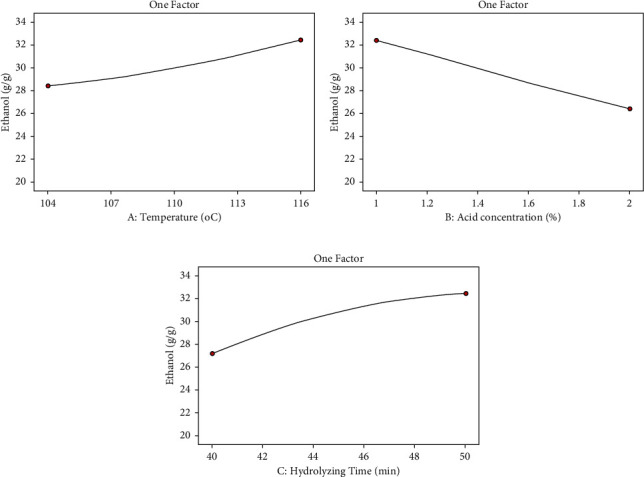
(a) The effects of temperature on the yield of ethanol. (b) The effects of acid concentration on the yield of ethanol. (c) The effects of hydrolyzing time on the yield of ethanol.

Single factors had their effects on the yield of ethanol as shown in Figures 3(a)–3(c). In Figure 3(a), the temperature had positive effects on the yield of ethanol. When the temperature increased, the yield of ethanol was increased proportionally. In Figure 3(b), the yield of ethanol was a negative relation with the acid concentration. For this experiment, when acid concentration was increased, the yield of ethanol was decreased. Finally, hydrolyzing time is also the independent factor that affects the yield of ethanol positively within a specified range. When the hydrolyzing time increased, the yield of ethanol was increased. However, these may not always be correct. The hydrolyzing time increased beyond the experimental data points; there may be other side products that were formed. For this study, the experiment was carried out within the temperature range of 104°C–116°C, the acid concentration within the range of 1%-2%, and hydrolyzing time of 40 minutes–50 minutes. The temperature, acid concentration, and hydrolyzing time were not in this range; the other relation may be happening.

### 3.6. The 3D Representation of Ethanol Yield

**Figure 4:**
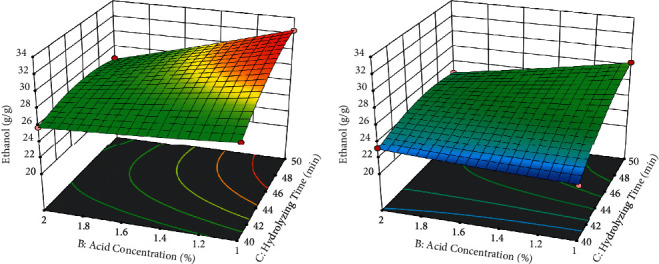
(a) The 3D response surface plot for the ethanol yield at a higher level of temperature (116°C). (b) The 3D response surface plot for the ethanol yield at a lower level of temperature (104°C).

From the 3D representation of Figure 4, the yield of ethanol was obtained at a higher level of temperature 116°C greater than at a lower level of 104°C. This shows that temperature has a significant and positive relation to the yield of ethanol obtained. In this condition, the temperature was maintained at a higher level and lower level, but the hydrolyzing time and acid concentration were variable. From the 3D representation of Figure 4(a) at 116°C of temperature when hydrolyzing time increased, the yield of ethanol was increased. However, when the acid concentration increased, the yield of ethanol was decreased. From Figure 4(b), the yield of ethanol was obtained at a lower level of temperature (104°C). Similarly, in Figure S1(a), the hydrolyzing time and acid concentration were varied on this surface response plot. From Figure S1(a), when the acid concentration was maintained to be constant at a lower level (1%), the yield of ethanol was increased proportionally with the increment of both temperatures and hydrolyzing time. The optimum results were obtained in this condition. Whereas when the acid concentration was maintained at a higher level, from Figure S1(b), the yield of ethanol was low. In the same way, from Figures S2(a) and S2(b), the hydrolyzing time was maintained constant at the higher and the lower level. In this condition, the yield of ethanol obtained was good at a higher level of hydrolyzing time than the lower level. This shows that the yield of ethanol was a positive relationship with the hydrolyzing time in this analysis. 3D representation of response surface methodology is to show the numerical values in a plot. For this study, the theoretical discussed points of each factor are represented in graphical patterns. The optimum yield of bioethanol was obtained at a temperature of 116°C, an acid concentration of 1.0%, and a hydrolyzing time of 50 minutes.

By the experimental analysis, the yield of ethanol was obtained within the range between 51.41% and 79.07%. In the study of [39], the optimum yield of ethanol obtained by acid hydrolysis was 78%; this was performed at a temperature of 100°C, an acid concentration of 0.4 M, and a hydrolyzing time of 1 hour. But in the current study, the optimum yield of ethanol obtained was 79.07%. This optimum yield was obtained at a temperature of 116°C, an acid concentration of 1%, and a time of 50 minutes. From another study performed by [40], the yield of ethanol obtained was 69% of these results compared with the current study; the current study had good results. For this study, the optimum results were obtained at a temperature of 116°C, at an acid concentration of 1%, and a hydrolyzing time of 50 minutes. To obtain good results, the temperature is the main factor for the hydrolysis process. This was determined by the sum square analyzed from the analysis of variance from the surface response. The second to the temperature hydrolyzing time was the significant factor that affects the yield of ethanol. Next to hydrolyzing time acid concentration was a significant factor. There are around 17 optimized results; from those, the higher desirability was selected. The optimized result of bioethanol produced was 29.9957; this was obtained at a temperature of 109.999°C, an acid concentration of 1.0000%, and a hydrolyzing time of 49.599 minutes, as shown in Table 3.

### 3.7. Bioethanol Properties

Bioethanol was characterized by its density, flammability, viscosity, boiling points, pH, alcohol content, and functional group analysis by using FTIR analysis. In this study, the bioethanol produced can be characterized and compared with another study. The bioethanol produced in this study had a density of 0.803 g/cm^3^; this density some deviation with ethanol commercially available. This was happening due to the presence of water within the ethanol produced. The viscosity of this bioethanol produced was 1.10 cP. The viscosity of bioethanol produced has a little variation with 98% pure bioethanol. The 98% pure bioethanol had a viscosity of 1.2 cP. The flammability of bioethanol is ranged from 12°C to 17°C; this was depending on the purity of ethanol. However, when the ethanol is highly flammable, its purity is also high, and when the ethanol is less flammable, its purity is low. Based on this, the flammability of bioethanol produced is 14°C which shows in a range between 12°C to 17°C. The ethanol produced had pH and boiling points of 6.65 and 80°C, respectively. The ethanol that has high purity had a pH of around 7 but the ethanol produced in this study has less pH than pure ethanol. This was happening because some acid residue was left during acid hydrolysis. The boiling point was also higher than the ethanol commercially available. This resulted in the water being not purified hundred percent.

#### 3.7.1. Functional Group Analysis of Ethanol

**Figure 5:**
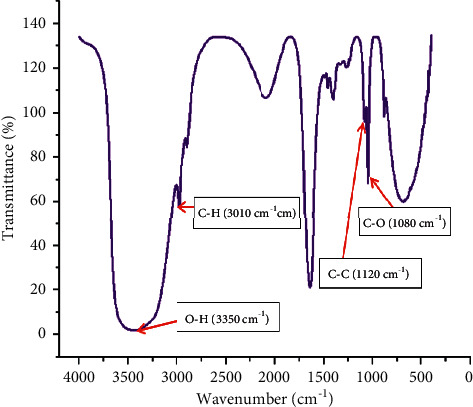
Functional group analysis for ethanol by using FTIR.

In the study of [41], the functional group of bioethanol was analyzed using Fourier-transform infrared spectroscopy (FTIR). The vibration of each bond stretch was obtained by using the absorbance and transmittance analyzed by the FTIR machine. The *X*-axis was wavenumber and *Y*-axis was the percent of transmittance. According to [42], the O-H stretch of hydrogen bond was ranged from 3500 to 3200 cm^−1^, the C-O stretch 1260 to 1050 cm^−1^, the C-H stretch from 3100 to 3000 cm^−1^, and the C-C bond stretched around 1100 cm^−1^. From Figure 5, the functional group of ethanol was analyzed by using FTIR data. The O-H stretch was observed at 3350 cm^−1^, the C-H stretch was observed at 3010 cm^−1^, for C-C stretch was observed at 1120 cm^−1^, and for C-O was observed at 1080 cm^−1^.

#### 3.7.2. Density and Purity of Ethanol

The density of ethanol was measured by using the above equipment and was obtained at 0.802 g/cm^3^. The density of pure ethanol was 0.789 g/cm³. Hence, ethanol produced from leftover injera waste was denser than pure ethanol. The difference between the densities of ethanol produced from waste food and pure ethanol was 1.5%. This difference was resulted due to the existence of water as a mixture of produced ethanol. The purity of ethanol was measured by using an alcohol meter. The ethanol produced had a purity of 70%. The purity of ethanol depends on the efficiency of distillation. In the first and second steps, the distillation process was carried out by using a heater as a heat source. In the first step, distillation process, the ethanol obtained was 35% and 65% was other impurities; in the second step, the amount of ethanol obtained was 40 percent and 60 percent was water. In the third and fourth steps, the water bath was used as a heat source. In this case, the temperature of the water bath was set at 79°C and the amount of ethanol obtained was 62 percent; in the fourth step, the temperature of the water bath was maintained at 78.4°C and the ethanol obtained was 70 percent.

#### 3.7.3. Viscosity, pH, Flammability, and Boiling Point of Ethanol

The pH of ethanol was measured by using a pH meter. Ethanol produced has a pH of 6.67, which was almost neutral. The pH of 100% pure ethanol has a pH of 7.33. Hence, the ethanol produced from waste food has been comparable pH with pure ethanol. This difference resulted due to the existence of a water mixture in the produced ethanol and there may be some acids present that were added during the hydrolysis process. The viscosity of ethanol was measured by using a viscometer and ethanol produced from leftover injera waste has a viscosity of 1.2 cP. This viscosity obtained was almost the same result compared to pure ethanol, pure ethanol has a viscosity of around 1.1, and this deviation has resulted due to impurities present within ethanol. The ethanol produced has a boiling point of 79°C, which had some deviation from pure ethanol. The bioethanol produced has the flammability of 15°C, but the pure ethanol has flammability ranging between 10 and 12°C. This deviation has occurred due to impurities or water present within the bioethanol. When the amount of water present in bioethanol has increased, the flammability of ethanol was decreased and the temperature for the flammability was increased. Pure ethanol has boiling points of 78°C, and this difference has happened due to some amount of water and impurities within liquid ethanol.

## 4. Conclusion

Response surface methodology (CCD) was used to analyze data in a better way. Based on this analysis, all independent factors had significant effects on the yield of ethanol. From those independent factors, the temperature has a significant effect on the yield of ethanol. The second factor that significantly affects the yield of ethanol was hydrolyzing time and the third was acid concentration. The optimum result obtained was 29.9 g/g. This was obtained at a temperature of 109.9°C, a time of 49.9 minutes, and acid concentration was 1.0%. Based on the current study, the response surface methodology with the employed central composite design provides acceptable results and the regression value *R*^2^ is 99.9%. This shows response surface methodology was employed; the good results were obtained for optimization of ethanol production. The method used in this research work was acid hydrolysis using sulfuric acid, which is very advantageous to decomposing the carbohydrate easily into fermentable sugar. However, the disadvantage of this method is that it is inevitable for the deterioration in the quality of the product. Ethanol produced in this experiment can be used as solvent for different chemicals, to wash and prevent laboratory pieces of equipment from contamination, and to blend with the fuel if it is further purified.

## Figures and Tables

**Table 1:** Coefficients in terms of coded factors.

Factor	Coefficient estimate	Df	Standard error	95% CI low	95% CI high	VIF
Intercept	26.75	1	0.0476	26.65	26.86	
A—temperature	1.70	1	0.0315	1.63	1.78	1.0000
B—acid concentration	−1.30	1	0.0316	−1.37	−1.23	1.0000
C—hydrolyzing time	1.67	1	0.0316	1.60	1.74	1.0000
AB	−0.5650	1	0.0412	−0.6569	−0.4731	1.0000
AC	−0.2200	1	0.0412	−0.3119	−0.1281	1.0000
BC	−1.19	1	0.0412	−1.28	−1.10	1.0000
A^2^	0.4419	1	0.0307	0.3736	0.5102	1.02
B^2^	0.1035	1	0.0308	0.0350	0.1721	1.02
C^2^	−1.02	1	0.0307	−1.09	−0.9561	1.02

**Table 2:** The proximate analysis obtained from laboratory.

Proximate analyses			
Analyses	Standard	% Wet bases	% Dry bases
Total moisture (wt. %)	ASTMD 3302	12	—
Volatile (wt. %)	ASTMD 3175	73.15	82.15
Ash (wt. %)	ASTMD 3174	0.93	1.1
Fixed carbon (wt. %)	ASTMD 3172	13.92	16.75

**Table 3:** The optimized result of bioethanol produced.

Number	Temperature	Acid concentration	Hydrolyzing time	Ethanol	Desirability	
1	109.9999	1.0000	49.5999	29.9957	0.8867	Selected

## Data Availability

The data used to support the findings of this study are available from the corresponding author upon request.

## Supplementary Materials

Supplementary Materials The supplementary files for both tables and figures have been provided as Table S1 to Table S3 for tables and Figure S1 to Figure S2 for figures, respectively.
