# Supplementary material for: Process Optimization for Acid Hydrolysis and Characterization of Bioethanol from Leftover Injera Waste by Using Response Surface Methodology: Central Composite Design
Source: Int J Anal Chem. 2022 Apr 6;2022:4809589. doi: 10.1155/2022/4809589 (PMC9007663; doi:10.1155/2022/4809589)
Supplement: Supplementary Materials — The supplementary files for both tables and figures have been provided as Table S1 to Table S3 for tables and Figure S1 to Figure S2 for figures, respectively. [file 4809589.f1.docx]

**Supplementary File**

1. **Supplementary Tables**

*Table S.1: Statistical data analyzed by ANOVA*

| Std. | run | Temperature (^o^ C) | Acid Concentration (%) | Hydrolyzing time (min) | The yield of Ethanol (g/g) |
| --- | --- | --- | --- | --- | --- |
| 1 | 1 | 104 | 1 | 40 | 22.1 |
| 2 | 14 | 116 | 1 | 40 | 27.23 |
| 3 | 8 | 104 | 2 | 40 | 23.2 |
| 4 | 5 | 116 | 2 | 40 | 25.82 |
| 5 | 12 | 104 | 1 | 50 | 28.42 |
| 6 | 9 | 116 | 1 | 50 | 32.42 |
| 7 | 13 | 104 | 2 | 50 | 24.51 |
| 8 | 18 | 116 | 2 | 50 | 26.5 |
| 9 | 16 | 99.9 | 1.5 | 45 | 25.16 |
| 10 | 2 | 120.1 | 1.5 | 45 | 30.84 |
| 11 | 7 | 110 | 0.66 | 45 | 29.32 |
| 12 | 10 | 110 | 2.3 | 45 | 24.76 |
| 13 | 3 | 110 | 1.5 | 36.6 | 21.08 |
| 14 | 15 | 110 | 1.5 | 53.5 | 26.62 |
| 15 | 11 | 110 | 1.5 | 45 | 26.82 |
| 16 | 20 | 110 | 1.5 | 45 | 26.79 |
| 17 | 6 | 110 | 1.5 | 45 | 26.74 |
| 18 | 17 | 110 | 1.5 | 45 | 26.81 |
| 19 | 4 | 110 | 1.5 | 45 | 26.83 |
| 20 | 19 | 110 | 1.5 | 45 | 26.52 |

*Table S.2: Analysis of variance for response surface of Ethanol yield obtained*

| source | Sum of squares | df | Mean square | F-value | P-value |
| --- | --- | --- | --- | --- | --- |
| Model | 135.14 | 9 | 15.02 | 1104.52 | < 0.0001 |
| A-Temperature | 39.73 | 1 | 39.73 | 2922.24 | < 0.0001 |
| B-Acid Concentration | 23.22 | 1 | 23.22 | 1708.23 | < 0.0001 |
| C-Hydrolyzing Time | 38.12 | 1 | 38.12 | 2804.18 | < 0.0001 |
| AB | 2.55 | 1 | 2.55 | 187.85 | < 0.0001 |
| AC | 0.3872 | 1 | 0.3872 | 28.48 | 0.0003 |
| BC | 11.33 | 1 | 11.33 | 833.33 | < 0.0001 |
| A² | 2.82 | 1 | 2.82 | 207.64 | < 0.0001 |
| B² | 0.1540 | 1 | 0.1540 | 11.33 | 0.0072 |
| C² | 15.13 | 1 | 15.13 | 1112.71 | < 0.0001 |
| Lack of Fit | 0.0665 | 5 | 0.0133 | 0.9565 | 0.5189 |

*Table S.3: Fit statistics obtained from ANOVA for Ethanol yield*

| Response  parameter | Std. Dev. | Mean | C.V% | R² | Adjusted R² | Predicted R² | Adeq. Precision | Model suggested |
| --- | --- | --- | --- | --- | --- | --- | --- | --- |
| Values | 0.1166 | 26.42 | 0.4412 | 0.9990 | 0.9981 | 0.9952 | 138.8042 | Quadratic |

1. **Supplementary Figures**


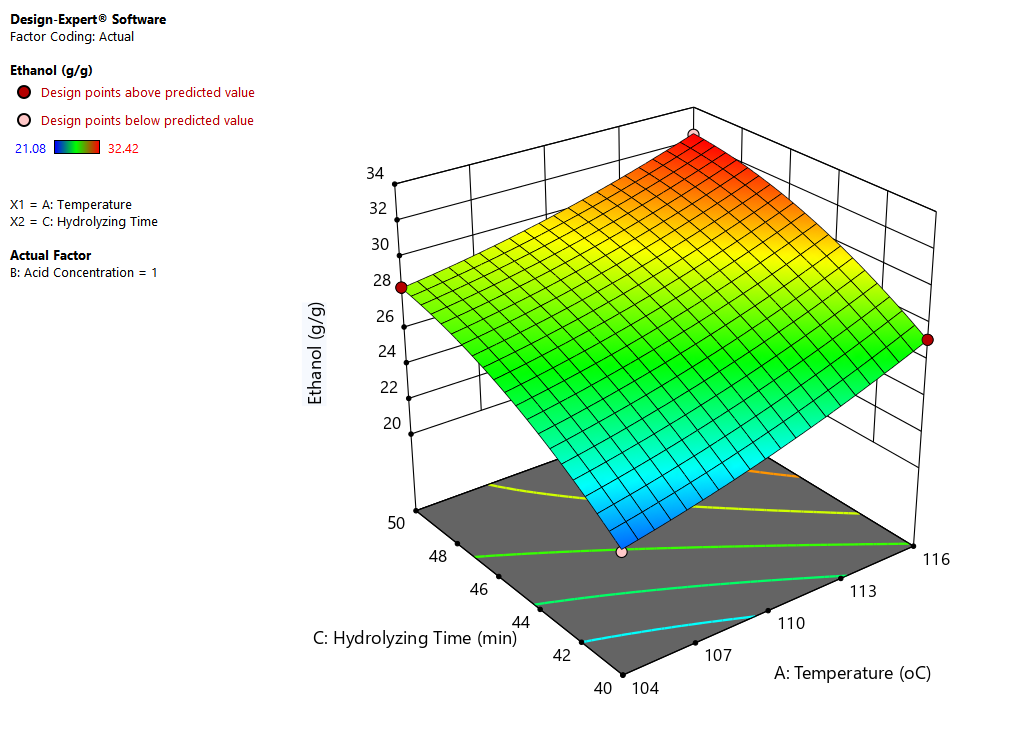


*Figure S.1 (a): The 3D response surface plot for the ethanol yield at a lower level of acid concentration (1%)*


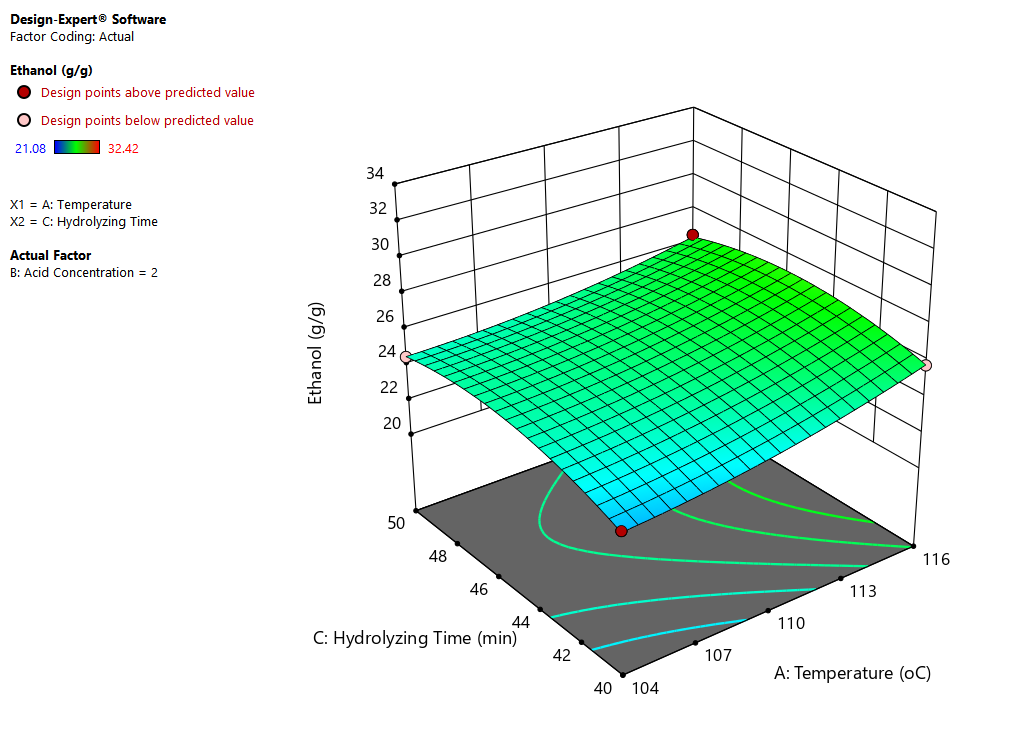


*Figure S.1 (b): The 3D response surface plot for the ethanol yield at a higher level of acid concentration (2%)*


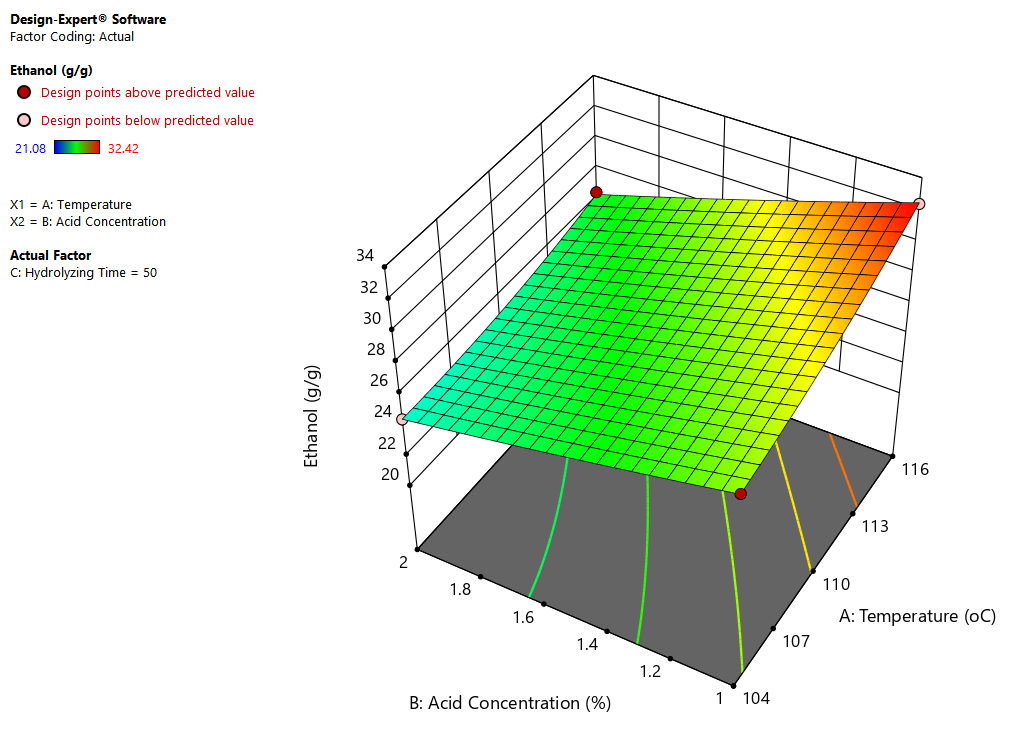


*Figure S.2 (a):* *The 3D response surface plot for the ethanol yield at a higher level of hydrolyzing time (50 min)*


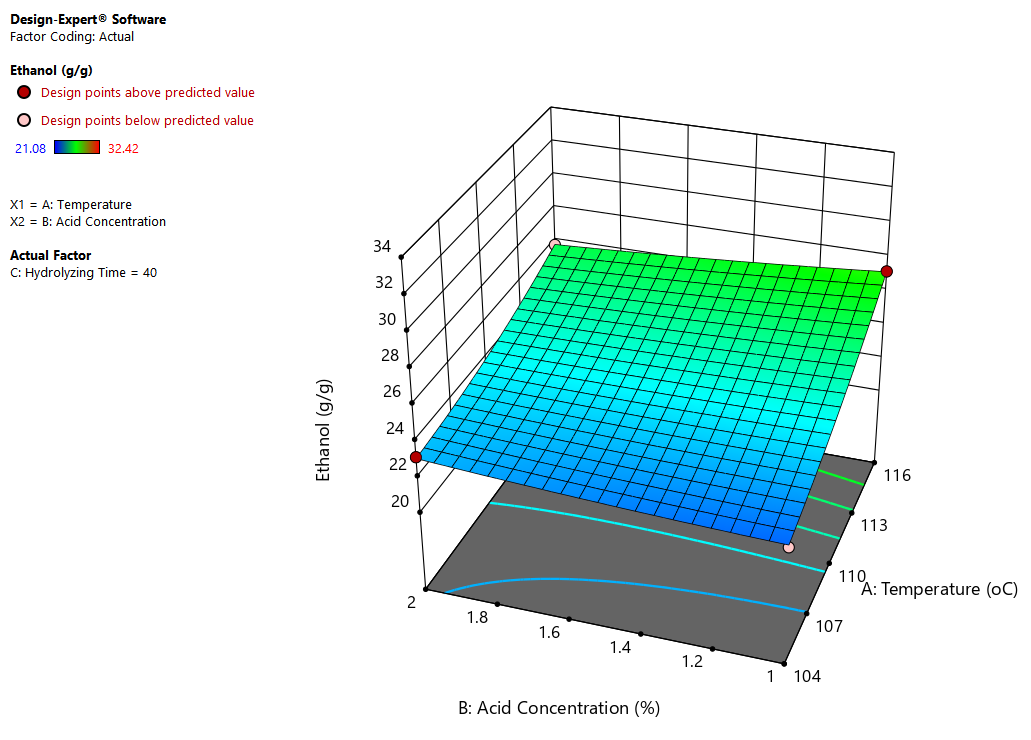


*Figure S.2 (b): The 3D response surface plot for the ethanol yield at a lower level of hydrolyzing time (40 min)*
